# Supplementary figures and images for: Knockdown of CDCA8 inhibits the proliferation and enhances the apoptosis of bladder cancer cells
Source: PeerJ. 2020 Apr 28;8:e9078. doi: 10.7717/peerj.9078 (PMC7194097; doi:10.7717/peerj.9078)

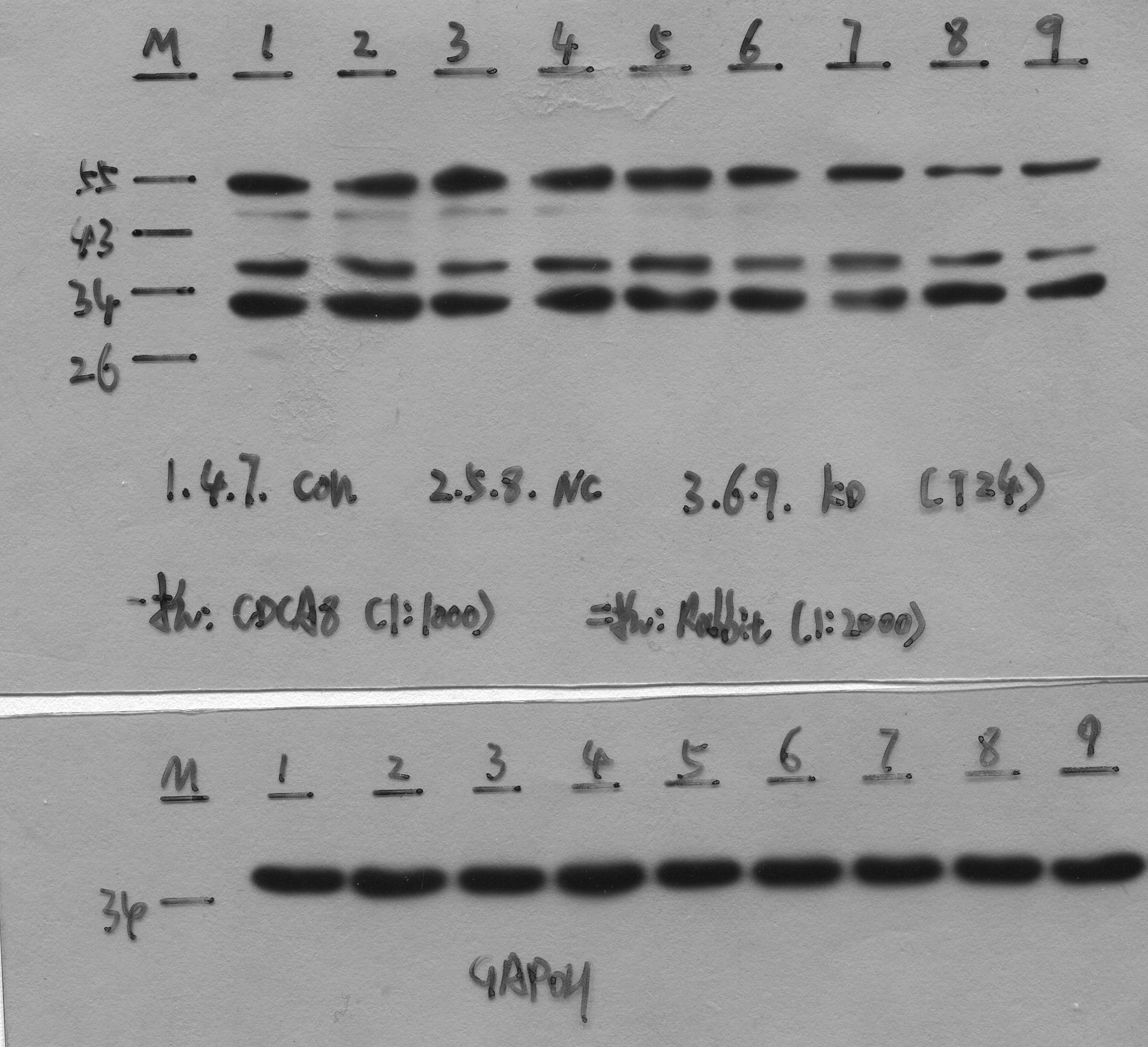

Supplement: Supplemental Information 2 [file peerj-08-9078-s002.zip › FileS1/WB.jpg]

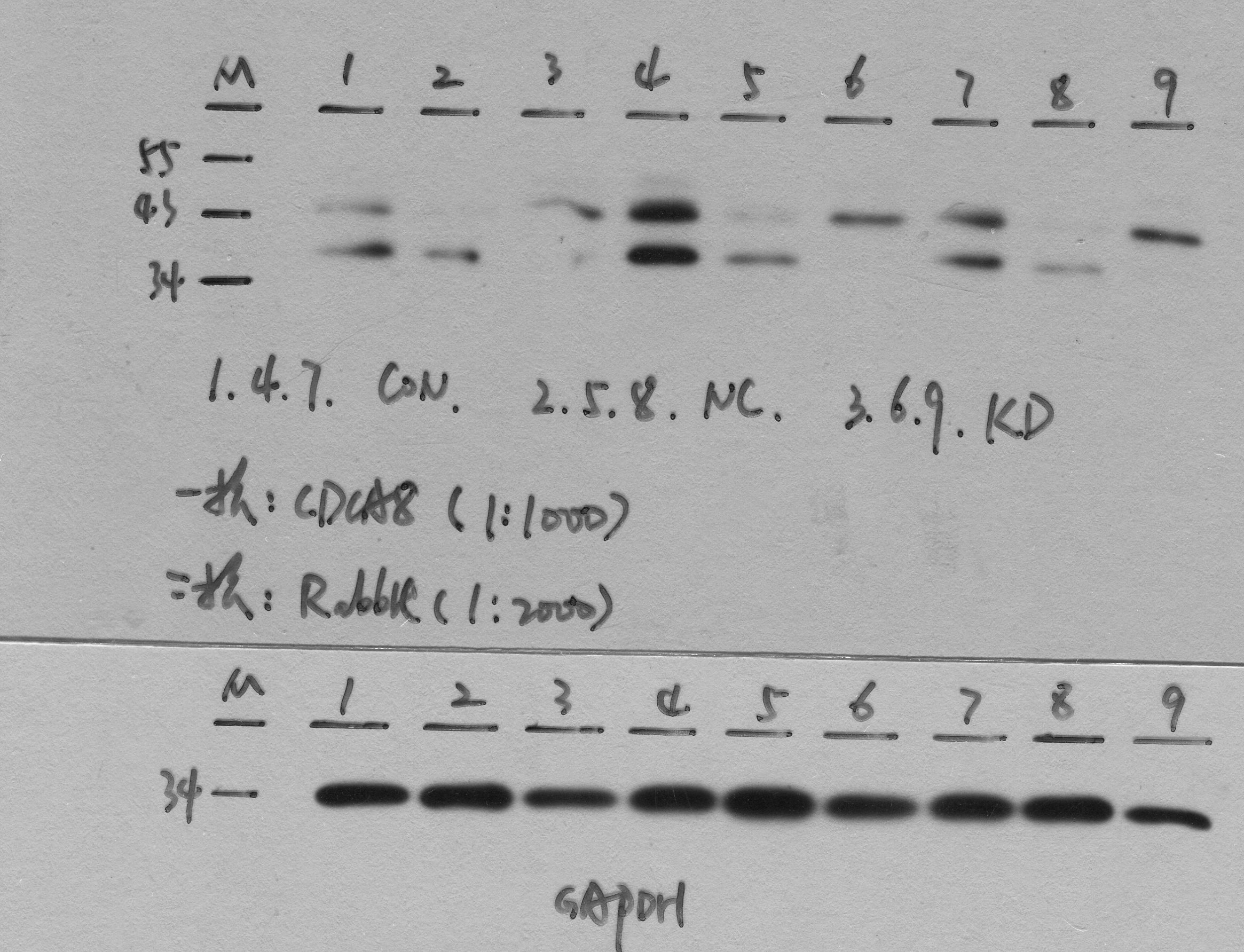

Supplement: Supplemental Information 2 [file peerj-08-9078-s002.zip › FileS1/WB_5637.jpg]
